# An Optimal Approach for Heart Sound Classification Using Grid Search in Hyperparameter Optimization of Machine Learning

**DOI:** 10.3390/bioengineering10010045

**Published:** 2022-12-29

**Authors:** Yunendah Nur Fuadah, Muhammad Adnan Pramudito, Ki Moo Lim

**Affiliations:** 1 Computational Medicine Lab, Department of IT Convergence Engineering, Kumoh National Institute of Technology, Gumi 39177, Republic of Korea; 2 School of Electrical Engineering, Telkom University, Bandung 40257, Indonesia; 3 Computational Medicine Lab, Department of Medical IT Convergence Engineering, Kumoh National Institute of Technology, Gumi 39177, Republic of Korea; 4 Meta Heart Co., Ltd., Gumi 39177, Republic of Korea

**Keywords:** heart sound signal, MFCC, grid search, k-nearest neighbor, artificial neural networks, random forest, support vector machine

## Abstract

Heart-sound auscultation is one of the most widely used approaches for detecting cardiovascular disorders. Diagnosing abnormalities of heart sound using a stethoscope depends on the physician’s skill and judgment. Several studies have shown promising results in automatically detecting cardiovascular disorders based on heart-sound signals. However, the accuracy performance needs to be enhanced as automated heart-sound classification aids in the early detection and prevention of the dangerous effects of cardiovascular problems. In this study, an optimal heart-sound classification method based on machine learning technologies for cardiovascular disease prediction is performed. It consists of three steps: pre-processing that sets the 5 s duration of the PhysioNet Challenge 2016 and 2022 datasets, feature extraction using Mel frequency cepstrum coefficients (MFCC), and classification using grid search for hyperparameter tuning of several classifier algorithms including k-nearest neighbor (K-NN), random forest (RF), artificial neural network (ANN), and support vector machine (SVM). The five-fold cross-validation was used to evaluate the performance of the proposed method. The best model obtained classification accuracy of 95.78% and 76.31%, which was assessed using PhysioNet Challenge 2016 and 2022, respectively. The findings demonstrate that the suggested approach obtained excellent classification results using PhysioNet Challenge 2016 and showed promising results using PhysioNet Challenge 2022. Therefore, the proposed method has been potentially developed as an additional tool to facilitate the medical practitioner in diagnosing the abnormality of the heart sound.

## 1. Introduction

Cardiovascular disease is one of the leading causes of mortality worldwide. In 2016, an estimated 17.9 million deaths occurred prematurely due to cardiovascular disease, accounting for 31% of all global deaths. Heart attacks and strokes are responsible for 85% of global deaths due to cardiovascular disease [1]. The high mortality rate is caused by cardiovascular problems that should be diagnosed early to avoid long-term complications and premature cardiac death.

Electrocardiogram (ECG) and phonocardiogram (PCG) are commonly used to diagnose cardiovascular disease. PCG is a graphical representation of heart-sound signals that can extract the heart-valve opening time more accurately than ECG signals [2]. As a result, heart-sound signals contain important physiological information about cardiac conditions that can be utilized to detect cardiac organ deformation and valve damage [3]. On the other hand, cardiac auscultation is determined by the physicians’ abilities and subjective experiences [4]. Therefore, an objective and automatic computer-assisted analysis of heart-sound signals is very important for the early diagnosis of cardiovascular diseases [5], which can potentially prevent premature death.

Automatic heart-sound classification is currently a promising research field based on signal processing and artificial intelligence approaches. It is reliable to screen or monitor for cardiac diseases in a wide range of clinical settings [6], allowing for the reduction of costly and laborious manual examinations [7]. Several studies have proposed machine learning algorithms to detect cardiovascular disorders based on heart-sound signals acquired using an electronic stethoscope or heart-sound signals acquired using Phonocardiography (PCG).

Barua et al. developed an accurate system for diagnosing valvular heart diseases (VHD) using a stethoscope sound dataset [8]. The handcrafted features from heart-sound signals were extracted using a dual symmetric tree pattern (DSTP) and multilevel discrete wavelet transform (DWT). Feature selection was performed using iterative neighborhood component analysis (INCA) to select 512 critical features. Furthermore, the Support Vector Machine (SVM) classifier model obtained an accuracy of 99.58% with 10-fold cross-validation (CV) and an accuracy of 99.84% with leave-one-subject-out (LOSO) CV in detecting VHD conditions.

Meanwhile, Tuncer et al. expanded the classification system to diagnose four conditions of valvular heart disease (VHD), including mitral valve prolapse, mitral regurgitation, mitral stenosis, and aortic stenosis based on phonocardiogram signals [9]. A novel multilevel feature generation network was proposed by combining the Petersen graph pattern (PGP) and the ten pooling (TEP) decomposition model. The feature selection process was performed using INCA, which will generate essential features as input to several classifier models, including k-nearest neighbor (K-NN), decision tree (DT), linear discriminant (LD), bagged tree (BT), and support vector machine (SVM). The highest performance obtained classification accuracy of 100% using K-NN. However, the implementation of INCA for feature selectors has a higher computational complexity.

Several studies proposed by Patidar et al. used tunable quality wavelet transform (TQWT) for heart-sound signal segmentation, noise removal, and classification. Constrained TQWT is an efficient method for automatic cardiac-sound signal segmentation [10]. Moreover, the vital information in the murmurs condition has been successfully extracted using constrained TQTW-based decomposition and reconstruction. Furthermore, Patidar et al. generated the novel raw feature set based on constrained TQTW, time domain representation, and Fourier–Bessel expansion of cardiac-sound signals [11]. After applying adaptive feature selection, the classification accuracy performance for heart-valve disorder identification obtained 94.01% and 93.53% using least squares support vector machine (LS-SVM) and short-time Fourier transform (STFT), respectively. Their study successfully decreased computational complexity by providing a lower dimensionality features vector.

In addition to valvular heart-disease detection, Patidar et al. developed an automatic diagnosis of septal defects based on TQWT, sum average magnitude difference function (SAMDF), and LS-SVM [12]. A novel feature set generated from TQWT-based SAMDF represents time-frequency properties of different types of murmurs. Furthermore, the proposed features are used as input to the LS-SVM classifier model with various kernel functions. The highest classification performance obtained an accuracy of 98.92%, a sensitivity of 98.80%, and a specificity of 99.29%. Involving the SAMDF-based feature set derived with TQTW can accurately represent the information of cardiac-sound signals for diagnosing septal defects. However, the validation of the system lacks rigorous k-fold cross-validation for training and testing due to the small dataset.

Sawant et al. proposed a novel method using the Fano-factor constrained TQTW for detecting the abnormality in heart-sound signals [13]. The TQTW decomposes the heart-sound signals into particular frequency bands, and the Fano-factor constraint is applied to select subbands for signal reconstruction. Furthermore, optimizing TQTW parameters and the Fano-factor threshold is performed using a genetic algorithm (GA) to provide optimal classification performance. A total of 315 features of the time and frequency domain are extracted from the signal reconstruction. The light gradient boosting model (Light GBM) learned the selected features and performed binary classification of heart-sound signals. The implementation of the synthetic minority over-sampling technique (SMOTE) to generate the balanced dataset provided a sensitivity of 86.32%, a specificity of 99.44%, and an overall score of 92.88%. The study limitations are that the model showed overfitting and could not generalize the unseen dataset.

Moreover, several studies implemented a deep learning model for heart-sound signals classification. Rubin et al. reported an 84% test accuracy using Mel frequency cepstral coefficients (MFCC) and a two-dimensional convolutional neural network (2D CNN) [14]. The preprocessing step selected a 3 s duration of heart-sound signals. After that, they extracted 13 MFCC features and converted these into 2D heat-map images as input for the 2D CNN. Nogueira et al. used 2D heat-map images of heart sound as input for the support vector machine (SVM) classifier algorithm. This method achieved an accuracy of 82.33% [15].

Meanwhile, in a study by Xiao et al., a validation accuracy of 93% was reported and verified via 10-fold cross-validation using a 1D convolutional neural network (1D CNN) [16]. The preprocessing step required resampling at 2000 Hz, removing noise using a band-pass filter and sliding window with 3 s patches and 1 s stride. The authors claimed that the proposed model provided low parameter consumption. As limitation of their study, the proposed method did not use test datasets that differed from the training and validation datasets. Li et al. proposed CNN as a classification method [17]. However, the method did not extract the features directly using CNN but required a separate feature-extraction process using multi-feature extraction. The proposed model was tested using 831 test datasets and reported an accuracy of 86.8% using 5-fold cross-validation. In a study by Khrisnan et al., a 6 s heart-sound recording duration was used as input for a feed-forward neural network (FNN) [18]. They verified the model using 5-fold cross-validation and reported an accuracy of 85.65% for test datasets.

Al-Naami et al. reported the highest accuracy at 89% using high-order spectral analysis and an adaptive neuro-fuzzy inference system (ANFIS) [19]. However, they only used 1837 heart-sound recordings from folder “a”, “b”, and “e” in PhysioNet Challenge 2016 datasets. Khan et al. reported a 91.39% accuracy using MFCC for feature extraction and LSTM as a classifier algorithm [20]. He et al. extracted 512 features using several feature extraction methods, such as the Hilbert envelope, homomorphic environment map, wavelet envelope, and power spectral density envelope as inputs for 1D CNN. This study reported an accuracy of 87.3% [21]. Jeong et al. used a 5 s heart-sound recording duration and removed datasets that were less than 5 s [22]. They applied a short-time Fourier transform (STFT) to transform to the time-frequency domain and generated spectrogram images of heart-sound signals as input for the 2D CNN. The proposed method obtained 91% accuracy for the 208 test datasets.

The previous studies commonly used a public dataset from PhysioNet Challenge 2016 for developing a system that can detect heart-sound abnormality. However, several studies used the PhysioNet Challenge 2022 dataset for heart-sound classification research. Monteiro et al. used a 4 s segment of heart-sound recording from the PhysioNet Challenge 2022 dataset [23]. They used signal features including homomorphic, Hilbert, power spectral density, and wavelet envelopes as input to Bidirectional Long Short-Term Memory (BiLSTM) neural network for murmur detection and clinical outcome prediction. After applying 5-fold cross-validation on the training set, the model was evaluated using the validation set. A murmur-weight accuracy of 0.751 was obtained to classify into three conditions: murmur present, murmur absent, and unknown.

Furthermore, Ballas et al. proposed a 5 s heart-sound signals segmentation and augmentation process in preprocessing steps [24]. Furthermore, the five convolutional layers extracted the information of the heart-sound signals that will be classified in the classification layers. The results reported a murmur-weighted accuracy score of 0.668 for the validation dataset and a murmur-weighted accuracy score of 0.737 for the hidden dataset. Based on these results, it is shown that the proposed model can generalize the unseen dataset for murmur detection. In addition, the weighted accuracy for clinical outcome prediction obtained 0.764 to classify normal and abnormal conditions.

There are several challenges in developing automated cardiac disorders using public datasets, such as the imbalance datasets of normal and abnormal conditions and the variation of data due to different recording procedures from clinical data around the world. The aforementioned studies reported promising results related to automated heart-sound detection performance. However, several studies showed overfitting due to the lack of a dataset and required high computational complexity to select the optimal parameters for providing the highest classification performance. Therefore, the accuracy must be enhanced because the early diagnosis of heart sounds is critical to saving patient life. In addition to the accuracy performance, low consumption parameters must be considered by researchers. Although the deep network provides good accuracy and can extract the information from raw signals directly, the complex architecture provides high consumption parameters and a long computational time.

Considering the limitations of the previous studies, we proposed an algorithm that can detect cardiac abnormalities based on heart-sound signals, improving accuracy and providing the optimal machine learning model. The Mel frequency cepstral coefficient (MFCC) as a feature extraction method has many advantages, such as capturing critical information in the audio signal and providing data as minimal as possible without losing information. This study used MFCC features directly as input to the machine learning model instead of converting MFCC features into 2D heat map images that required high computational complexity.

The performance of several machine learning models, including k-nearest neighbor (K-NN), random forest (RF), artificial neural network (ANN), and support vector machine (SVM), will be evaluated using two public datasets from PhysioNet Challenge 2016 and 2022 to provide the optimal approach in classifying heart-sound signal conditions. However, most machine learning models will only obtain optimal performance if model parameters are tuned correctly. Hyperparameter tuning is discovering the set of hyperparameter values expected to create the most accurate prediction model among all analyzed sets of hyperparameter values. Manually optimizing the machine learning parameters is time-consuming, particularly when the machine learning algorithm includes many parameters. Moreover, appropriate parameter settings will lead to better classification performance. Therefore, this study addressed this critical gap by applying the grid search method to select the best parameter of each machine learning model automatically.

## 2. Materials and Methods

**Figure 1:**
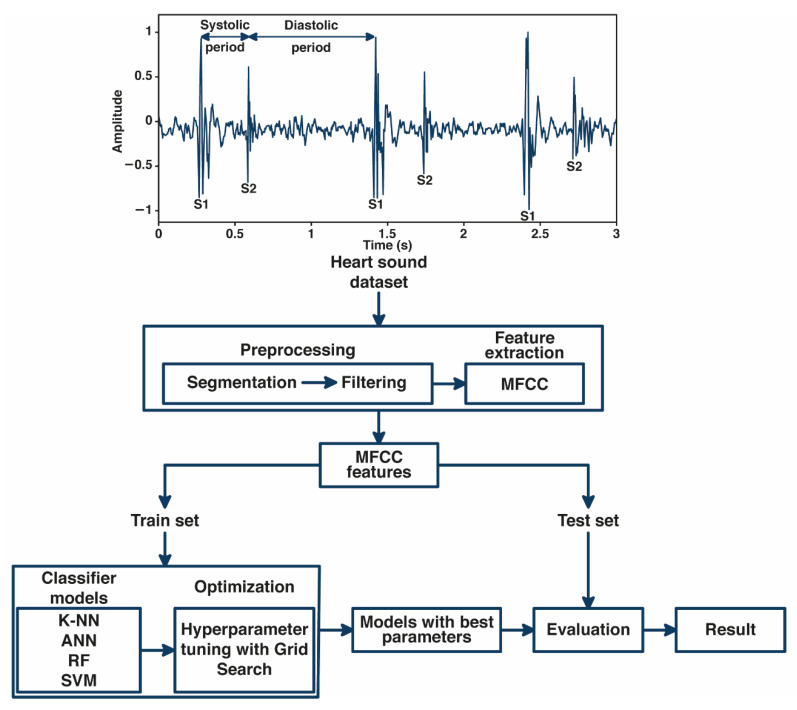
General block diagram system.

In this study, we developed an automated classification of normal and abnormal heart-sound signals based on MFCC features and machine learning, as shown in Figure 1. The fundamental heart-sound signal (FHS) is represented by the first heart sound (S1) and the second heart sound (S2). S1 often occurs during the onset of isovolumetric ventricular contraction. Meanwhile, S2 occurs when the aortic and pulmonic valves close at the onset of diastole. The period from S1 to S2 is called the systolic period, and the period from S2 to S1 is called the diastolic period.

The heart-sound signal was segmented into 5 s segments and filtered to remove noises in the processing. MFCC, as a feature extraction method, extracted the cepstral coefficients from heart-sound signals to be used as input to the machine learning model. In classification, we implemented Grid Search for tuning the hyperparameter of several machine learning classifier models, including K-NN, ANN, RF, and SVM. The performance of each classifier model was evaluated by analyzing the results based on accuracy, precision, recall, F1-score, and AUC score.

### 2.1. Database and Preprocessing

This study used two heart-sound signals public datasets collected from the PhysioNet Challenge 2016 and 2022 datasets. The PhysioNet Challenge 2016 is the public dataset widely used in previous studies for heart-sound analysis [25]. The datasets consisted of six folders from “a” to “f” with a total of 3160 heart-sound recordings; 2495 are normal conditions, and 665 are abnormal conditions such as mitral regurgitation, aortic stenosis, and valvular surgery [18]. The duration of each heart sound varied from 5 to 120 s with a 2000 Hz sampling frequency. However, recent studies have claimed that 5 s is the minimum duration required to detect heart-sound abnormalities. Therefore, this study divided the heart sound into short recordings of 5 s using time-series segmentation. On the other hand, this process can expand the heart-sound datasets by generating 16,251 more datasets, consisting of 12,418 normal and 3833 abnormal conditions.

Meanwhile, the PhysioNet Challenge dataset consists of heart-sound recordings with a total number of 5272 recordings that are collected from 1568 subjects in four auscultation locations, including pulmonary valve point (PV), tricuspid valve point (TV), aortic valve point (AV), and mitral valve point (MV) [26]. Since the duration of each heart-sound recording varied, we segmented the heart-sound recording into short recordings with duration 5 s. Therefore, we generated 3880 heart-sound signals of normal and 4201 heart-sound signals of abnormal conditions. Furthermore, we applied a Butterworth band pass filter with frequencies cut off 0.025–0.4 Hz to remove noises for each dataset.

### 2.2. Feature Extraction Using MFCC

**Figure 2:**
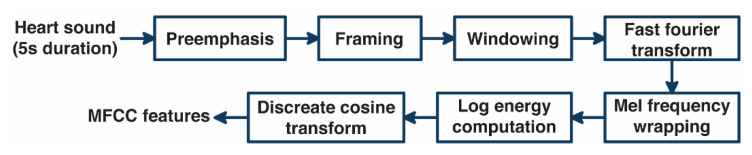
Block diagram of MFCC method.

The feature extraction of heart sound using MFCC consists of several processes [15], as shown in Figure 2, namely pre-emphasis, framing, windowing, and fast Fourier transform (FFT) to transform the heart-sound signal from the time domain to the frequency domain, which is then transformed to the Mel frequency domain, generating the Mel filter bank. The Mel filter bank value shows the amount of energy in the frequency range of each Mel filter. A non-linear transformation is performed to take the natural logarithmic value of each Mel filter. In the last process, the discrete cosine transform (DCT) returns the heart-sound signal to the time domain and produces MFCC features as input to the ANN. The number of widely used coefficients is 13–42 MFCC features. However, Hasan et al. claimed that the optimum number of MFCCs should be 25 [27]. Therefore, the number of MFCC features used in this study was 13, 25, and 42.

### 2.3. The Grid Search Based of Machine Learning Optimization

Grid Search (GS) is the most straightforward optimization technique for hyperparameter tuning in machine learning. GS technique will execute each possible combination of all hyperparameter values into grid configuration [28,29,30]. Furthermore, the GS will train the model of all possible hyperparameter combinations in Cartesian products from a finite set of values defined by the user. Each combination’s performance is assessed using a held-out validation set on the training set. The GS algorithm then generates the configurations that provide the best performance during the validation process. The model then uses the best hyperparameter values determined by the grid search. The GS was implemented and ensured that the best hyperparameters were generated. However, since the number of evaluations grows exponentially as the frequency of the hyperparameters rises, GS is ineffective in the configuration space of high-dimensionality hyperparameters. Suppose that k parameters are assigned with n distinct values; Grid Search’s complexity is predicted to increase exponentially at an O(nk) rate. Therefore, the hyperparameter setting should be constrained to make GS efficient as an optimization approach.

**Figure 3:**
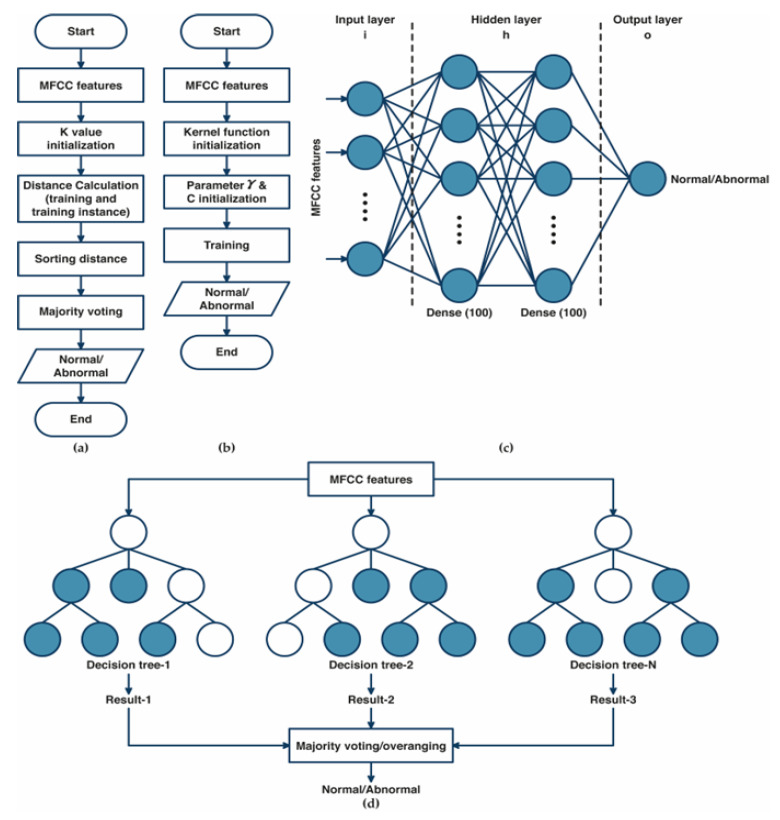
(**a**) The diagram of the k-nearest neighbor classifier algorithm; (**b**) The diagram of the support vector machine classifier algorithm; (**c**) The architecture of the artificial neural network algorithm; (**d**) The topology of random forest classifier algorithm.

The algorithm of k-nearest neighbor (K-NN) classifier models is illustrated in Figure 3a. The training data were projected into a multidimensional space, with each dimension representing the features extracted from the training data [31,32]. The algorithm of the training process included storing feature vectors and labels from the training data. Meanwhile, the unlabeled testing data were assigned to the label of its k closest neighbors during the classification process. Distance metrics such as Euclidean, Chebyshev, and Minkowski were used to measure the distance between two feature vector positions of the training and the testing data in multidimensional space. The MFCC features from heart-sound signals were classified as normal and abnormal conditions using majority votes based on the labels of its k-nearest neighbors. The optimization of K-NN was performed by hyperparameter tuning using grid search. The best parameter, as well as the best k value selection for varied values of k (k = 1, 3, 5, 7,…, 31), the distance matrix, including Euclidean, Minkowski, and Chebyshev, were selected throughout the optimization process using the grid search method [32].

The SVM algorithm’s main workflow is shown in Figure 3b. A kernel mapping function was used to convert the input feature vector into higher-dimensional feature spaces [33,34]. The SVM algorithm starts with the initialization of the kernel function, then the initialization of γ and C parameters. In this study, we tried three kernel functions—linear, radial basis functions (RBF), and polynomial. The selection of γ parameters (from 0.1 to 0.001) and the regularization of C parameter selection for SVM (from 1 to 1 × 10^3^) was evaluated as part of the SVM algorithm’s optimization process using the grid search method.

An ANN is a fully connected structure comprising three layers of its architecture [27]: input, hidden, and output layers (Figure 3c). The input layer acquires input from external resources. The feature extraction results of MFCC features were assigned as input for the ANN architecture. The hidden layers process the input from the previous layer and transfer the result to the output nodes. In this study, the hidden layers that were used in the ANN consisted of two hidden layers with 100 nodes for each hidden layer. The Rel-U activation function was applied to the hidden layer, and a sigmoid activation function was applied to the output layer, consisting of two nodes representing normal and abnormal conditions. The optimization of ANN was performed using a grid search to select the best optimizer among Adam, Nadam, SGD, and RMSprop optimizers and the best learning rate (from 0.1 to 0.001).

Figure 3d illustrates the structure of the random forest (RF) classifier. RF is a group of decision tree classifier algorithms that produces excellent performance instead of using a single decision tree [35,36]. RF provides several advantages, including fast computation times, insensitivity to noise in the dataset, and reduced overfitting. All the trees participate in the classification process by voting for their classes, and RF classifies the input according to the majority vote results. The grid search method determines the optimum number of trees (50, 100, 150, and 200) and the best criterion (gini and entropy) that yields the best performance result as the RF’s optimal parameter.

### 2.4. System Performance

The parameters for measuring the system’s efficacy in classifying heart sound, including the accuracy, weighted accuracy, and $F_{\beta }$-score of the system, were calculated using the following equation [28,37]:

(1) $$Accuracy=\frac{TP+TN}{TP+FP+TN+FN}$$

(2) $$Weighted\;Accuracy=\frac{5TP+TN}{5\left(TP+FN\right)+\left(FP+TN\right)}$$

(3) $$Precision=\frac{TP}{TP+FP}$$

(4) $$Recall=\frac{TP}{TP+FN}$$

(5) $$F_{\beta }-score=\left(1+\beta^{2}\right)\cdot \frac{Recall\cdot Precision}{Recall+\left(\beta^{2}\;\cdot \;Precision\right)}$$

A true positive (*TP*) is a result whereby the model accurately predicts the abnormal condition as the abnormal condition. Conversely, a true negative (*TN*) results in the model accurately predicting the normal condition as the normal condition (negative class). A false positive (*FP*) is a result whereby data are normal but misclassified as abnormal. Meanwhile, a false negative (*FN*) results in abnormal data but is misclassified as normal [28]. As shown in Equation (1), accuracy measures the number of data correctly classified according to their class divided with the whole test dataset. In addition, the weighted accuracy metric for the clinical outcome abnormal and normal is presented in Equation (2) based on the scoring metric from PhysioNet Challenge 2022 [37]. As shown in Equation (3), the $F_{\beta }$-*score* is commonly used to evaluate the system performance using an imbalanced dataset. In this study, we set β value equal to 2 (β > 1), since we are more concerned with recall than precision value.

Furthermore, this study also provided the receiver operating characteristic (ROC) curve that shows the true positive rate (TPR) against the false positive rate (FPR). As a summary of the ROC curve, the area under the curve (AUC) evaluates a classifier’s ability to differentiate between classes. Meanwhile, the area under the precision-recall curve (AUPRC) is essentially used to evaluate the system’s performance using an imbalanced dataset where recall value is more important than precision value to be analyzed.

## 3. Results

We applied the grid search method with 5-fold cross-validation using two public datasets: PhysioNet Challenge 2016 and 2022. For the PhysioNet Challenge 2016 dataset, we used 13,000 heart-sound signal segments of training data to select the best parameter of the classifier model. The proposed model was assessed using 3251 test data, including 2473 signals of normal and 778 signals of abnormal conditions. Meanwhile, for PhysioNet Challenge 2022, we used 6464 heart-sound signal segments of training data that will be trained to determine the best parameter of each classifier model. The model was evaluated using 1617 test data, including 757 signals of normal conditions and 860 signals of abnormal conditions. After conducting several simulations, the classification performance for PhysioNet Challenge 2016 and 2022 datasets is presented in Table 1.

The optimal k-values (k = 1, 3, 5, 7,…, 31) and distance matrices (i.e., Euclidean, Chebyshev, and Minkowski) for the K-NN classifier algorithm were chosen using the grid search approach. For both datasets, the Minkowski distance with an optimal value of k = 1 was chosen as the K-NN algorithm’s best parameter since it obtained the optimal classification performance. The system performance using the PhysioNet Challenge 2016 dataset obtained the highest classification performance of test data with an accuracy of 95.78%, weighted accuracy score of 0.93, $F_{\beta }-score$ of 0.96, AUC score 0.95, and AUPRC score 0.92 using 42 MFCC features. Meanwhile, the system performance using the PhysioNet Challenge 2022 dataset obtained the highest classification performance, including an accuracy of 76.31%, weighted accuracy score of 0.78, $F_{\beta }$-*score* of 0.75, AUC scores of 0.75, and AUPRC score of 0.81 using 42 MFCC features.

For the ANN classifier model, the grid search method selected the Adam optimizer with a learning rate of 0.001, epoch 1000, and batch size 64 as the best parameter. Therefore, we used these parameters for training the ANN model. The highest classification performance of test data from PhysioNet Challenge 2016 obtained 94.16% classification accuracy, weighted accuracy score of 0.91, $F_{\beta }-score$ of 0.94, AUC score of 0.92, and AUPRC score of 0.91 using 42 MFCC features. Meanwhile, the highest classification performance of test data from PhysioNet Challenge 2022 obtained an accuracy of 65.18%, weighted accuracy of 0.68, $F_{\beta }-score$ of 0.69, AUC score of 65, and AUPRC score of 0.76 using 25 MFCC features.

Furthermore, the grid search method selected “gini” as the best criterion for the RF classifier model, with 150 trees as the optimal parameter. The highest classification performance of test data from PhysioNet Challenge 2016 obtained an accuracy of 93.47%, weighted accuracy of 0.91, $F_{\beta }-score$ of 0.93, AUC score of 0.90, and AUPRC score of 0.88 using 42 MFCC features. In addition, the highest classification performance of test data from the PhysioNet Challenge 2022 dataset using the RF model obtained an accuracy of 68.76%, weighted accuracy of 0.69, $F_{\beta }-score$ of 0.69, AUC score of 0.68, and AUPRC score of 74 using 25 MFCC features.

Meanwhile, for the SVM classifier model, the grid search method selected a linear kernel with C = 1000 and γ = 0.01 as the best parameter. The highest classification performance of test data from PhysioNet Challenge 2016 obtained an accuracy of 86.19%, weighted accuracy of 0.79, $F_{\beta }-score$ of 0.85, AUC score of 0.77, and AUPRC score of 0.71. In addition, the highest classification performance of test data from PhysioNet Challenge 2022 obtained an accuracy of 58.81%, weighted accuracy of 0.59, $F_{\beta }-score$ of 0.58, AUC score of 0.58, and AUPRC score of 0.64 using 13 MFCC features.

**Figure 4:**
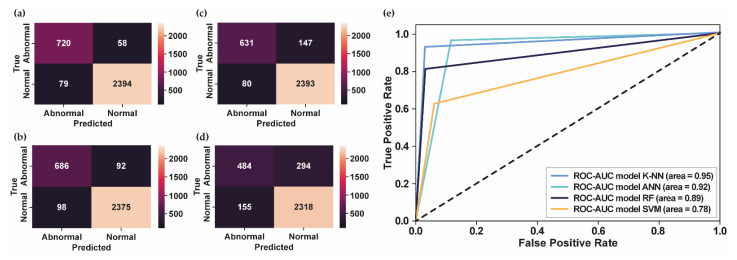
**The confusion matrix and ROC curve for PhysioNet Challenge 2016.** (**a**) The confusion matrix of test datasets using K-NN; (**b**) The confusion matrix of test datasets using ANN; (**c**) The confusion matrix of test datasets using RF; (**d**) The confusion matrix of test datasets using SVM; (**e**) The ROC curve of several classifier models.

According to the results, the number of MFCC features as input to the classifier models plays a vital role in obtaining the best system performance. For the PhysioNet Challenge 2016 dataset, 42 MFCC features that were input into classifier models provided the best performance compared to 13 and 25 MFCC features, as shown in Table 1. Figure 4 shows the comparative performance of several classifier models using 42 MFCC features as input.

Even though the result using K-NN is still affected by false detection, as shown in Figure 4a, most of the test datasets were successfully classified according to their class, followed by ANN in Figure 4b and RF in Figure 4c. Meanwhile, the number of false detections for normal and abnormal conditions for the SVM classifier, as shown in Figure 4d, is still high compared to the other classifier models. Figure 4e presents the ROC curve and the AUC score of each model. The greater the AUC value, the better the model can distinguish between normal and abnormal heart sounds. As shown in Figure 4e, the highest AUC score was 0.95, obtained using K-NN, followed by ANN, RF, and SVM, with AUC scores of 0.92, 0.89, and 0.78, respectively. In addition, the K-NN classifier model provided the highest results of $\;F_{\beta }-score$ and AUPRC score, which showed that the proposed model successfully detected the abnormality in heart-sound signals. Therefore, the proposed model can overcome the imbalanced datasets of PhysioNet Challenge 2016 and generalize the test datasets well.

**Figure 5:**
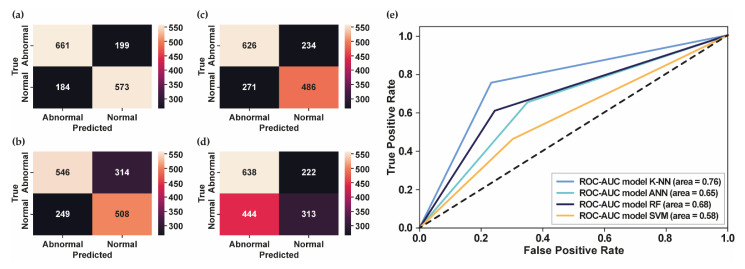
**The confusion matrix and the ROC curve for PhysioNet Challenge 2022.** (**a**) The confusion matrix of test datasets using K-NN; (**b**) The confusion matrix of test datasets using ANN; (**c**) The confusion matrix of test datasets using RF; (**d**) The confusion matrix of test datasets using SVM; (**e**) The ROC curve of several classifier models.

Figure 5a–d shows the confusion matrix for each classifier model using PhysioNet Challenge 2022. Meanwhile, Figure 5e presents the ROC curve of each classifier model that showed the highest AUC score obtained by K-NN using 42 MFCC features, followed by the RF model using 25 MFCC features, the ANN model using 25 MFCC features, and the SVM model using 13 MFCC features. In contrast with the PhysioNet Challenge 2016 dataset, which has an imbalanced dataset, the PhysioNet Challenge 2022 dataset has a balanced dataset. According to the confusion matrix in Figure 5a, the model showed promising results in accurately detecting the abnormal data as abnormal conditions. Moreover, the AUPRC score of 0.81 showed that the proposed model could detect abnormalities in heart-sound signals. However, the performance needs to be improved to provide better classification performance.

## 4. Discussion

In this study, an automated classification of cardiovascular disorders based on heart sound was proposed using MFCC as a feature extraction method and K-NN, ANN, RF, and SVM as a classification method. The two public datasets from PhysioNet Challenge 2016 and 2022 were used to develop and evaluate the heart-sound classification system. The results obtained in this study are superior to those of the latest studies that involve the same dataset, as shown in Table 2.

In terms of preprocessing, we discussed the selection of the heart-sound duration. An optimized heart-sound duration can obtain the best performance accuracy. Khrisnan et al. and Liu et al. suggested that a data record of at least 5 s is required for the reliable detection of heart abnormalities. Therefore, the duration was set to 5 s, similar to previous studies [19,22]. Compared with the previous studies that used the same duration length to detect abnormalities in heart-sound signals, the proposed model obtained superior performance accuracy, as shown in Table 2.

The number of MFCC feature selections for feature extraction was determined, as shown in Table 1. The best performance was achieved using 42 MFCC features. Instead of directly using the MFCC features, previous studies used the heat-map image of MFCC features as input for a 2D CNN, requiring a long computational time [14,15]. Other studies used various methods to extract the features from heart-sound signals; for example, Xiao et al. used a 1D time series consisting of 6000 data points as input for ID-CNN. Li et al. used multi-feature extraction, including 20 features in the time domain, 12 amplitude features, 42 features in the energy domain, 16 features in the higher order statistic domains, 65 features in the cepstrum domain, 308 features in the frequency domain, four features in cyclostationarity domain, and 10 features in the entropy domain. Therefore, 497 features were obtained and served as input for 1D CNN. He et al. extracted 512 features from the Hilbert envelope, homomorphic environment map, wavelet envelope, and power spectral density envelope.

Meanwhile, Jeong et al. used STFT as a feature extraction method and used 217 × 334 pixel heart-sound spectrogram images as input for 2D CNN. Compared with the previous studies, this study successfully extracted 42 MFCC features that provided fewer data points as feature results. However, the extracted features can represent normal and abnormal heart-sound signals with reduced computational time.

In most studies on heart-sound classification, machine learning and deep learning algorithms have the same goal of providing high accuracy and low parameter consumption, thereby reducing computational time. Deep learning is reliable for automatically extracting the heart-sound characteristics from the raw data signals. On the other hand, the training process takes a long time and requires a larger dataset to avoid overfitting. Moreover, a complex architecture will produce a more significant number of parameters and require a long computation time.

As shown in Table 2, the proposed K-NN model with 42 MFCC features outperformed the previous studies, which used the same dataset from PhysioNet Challenge 2016 by providing the highest classification performance of test data, including accuracy of 95.75%, weighted accuracy score of 0.93, $F_{\beta }$-*score* of 0.96, UC scores of 0.95, and AUPRC scores of 0.92. The highest performance results with the high value of the accuracy, weighted accuracy, $F_{\beta }$-*score*, AUC score, and AUPRC score value showed that the proposed model has a good classification performance, even though the dataset was imbalanced. Moreover, the high value of the $F_{\beta }$-*score* and AUPRC score showed the model’s ability to detect abnormal data without incorrectly detecting normal data as abnormal (perfect recall). Therefore, we can conclude that the proposed model can overcome the imbalanced dataset problem.

Furthermore, the proposed K-NN model with 42 MFCC features showed acceptable results compared with the latest studies that used the same dataset from PhysioNet Challenge 2022 by providing an accuracy of 76.31%, weighted accuracy score of 0.78, $F_{\beta }$-*score* of 0.75, AUC score of 0.75, and AUPRC of score 0.81. As shown in Table 2, Monteiro et al. obtained a murmur-weighted accuracy score of 0.751 in classifying present murmur, absent murmur, and unknown conditions [23]. Meanwhile, Ballas et al. obtained a murmur-weighted accuracy score of 0.731 and a clinical outcome-weighted score of 0.764 [24]. According to these comparison results, our proposed model showed promising results in detecting the abnormality in heart-sound signals using the PhysioNet Challenge 2022 dataset.

The main advantage of this study is providing the optimal machine learning algorithm with hyperparameter tuning using a grid-search method. The implementation of grid search reduced the computational time compared to manually selecting the best parameter of each classifier model. Moreover, the characteristics of normal and abnormal heart-sound signals were captured by MFCC as a feature extraction method and successfully identified using K-NN as a simple machine learning algorithm. The low parameter consumption resulted in fast computational time for screening the abnormality of heart sounds for cardiovascular disorder detection. Therefore, it helps the cardiologist provide a more efficient and effective treatment approach for the patients. However, there are several limitations of this study. The proposed model is still limited to classifying two conditions of heart-sound signals, which are normal and abnormal. Moreover, the classification performance still needs to be improved, especially for the PhysioNet Challenge 2022 dataset. Therefore, we need further research in developing the system to solve multiclass heart-sound classification problems for clinical feasibility in diagnosing cardiac abnormality based on the heart-sound signals.

## Figures and Tables

**Table 1:** The classification performance using several classifier models.

Models	MFCC Features	PhysioNet Challenge 2016					PhysioNet Challenge 2022				
		Acc	W.acc	$F_{\beta }$-Score	AUC	AUPRC	Acc	W.acc	$F_{\beta }$-Score	AUC	AUPRC
K-NN	13	93.44%	0.90	0.93	0.91	0.88	68.76%	0.70	0.69	0.69	0.75
	25	94.80%	0.91	0.95	0.93	0.90	71.98%	0.73	0.72	0.72	0.77
	42	95.78%	0.93	0.96	0.95	0.92	76.31%	0.78	0.76	0.76	0.81
ANN	13	92.43%	0.88	0.93	0.90	0.88	63.38%	0.65	0.65	0.63	0.75
	25	92.77%	0.90	0.94	0.91	0.90	65.18%	0.68	0.69	0.65	0.76
	42	94.16%	0.91	0.94	0.92	0.91	64.81%	0.67	0.68	0.65	0.76
RF	13	92.83%	0.91	0.93	0.90	0.88	64.81%	0.66	0.65	0.64	0.71
	25	93.35%	0.91	0.93	0.90	0.88	68.76%	0.69	0.69	0.68	0.74
	42	93.47%	0.91	0.93	0.90	0.88	68.46%	0.68	0.69	0.68	0.74
SVM	13	83.97%	0.77	0.84	0.75	0.69	58.81%	0.59	0.58	0.58	0.64
	25	84.44%	0.78	0.84	0.75	0.69	58.44%	0.58	0.58	0.58	0.63
	42	86.19%	0.79	0.85	0.77	0.71	58.62%	0.59	0.58	0.58	0.64

**Table 2:** Performance comparison with previous studies.

Authors	Preprocessing	Feature Extraction	Classifier	Accuracy
Rubin et al. (2016) [14]	Heart-sound segmentation using springer algorithm, selected 3 s duration of heart sound	Extracted 13 MFCC features, converted into 2D feature maps	2D CNN	84%
Nogueira et al. (2019) [15]	Logistic regression-HSMM-based heart-sound segmentation	Extracted MFCC features, converted into 2D feature maps	SVM	82.33%
Xiao et al. (2019) [16]	Resample 2000 Hz, BPF, sliding window with 3 s patches and 1 s stride	1D time series signal	1D CNN	93%
Li et al. (2020) [17]	HPF and HSMM	Multi feature extraction	1D CNN	86.80%
Khrisnan et al. (2020) [18]	Down-sampled to 500 Hz and selected 6 s heart-sound duration	1D time series signal	FNN	85.65%
Al-Naami et al. (2020) [19]	Band pass notch filter, BPF, and selected 5 s heart-sound duration	Higher order statistics	ANFIS	89%
Khan et al. (2021) [20]	Logistic regression-HSMM-based heart-sound segmentation	MFCC features	LSTM	91.39%
He et al. (2021) [21]	Normalization, HPF and LPF	Hilbert envelope, homomorphic environment map, wavelet envelope, and power spectral density envelope (512 data points)	1D CNN	87.30%
Jeong et al. (2021) [22]	BPF, selected 5 s heart-sound duration	STFT	CNN	91%
Monteiro et al. (2022) [23]	Selected 4 s heart-sound duration	Homomorphic, Hilbert, power spectral density, and wavelet envelopes	BiLSTM	75.1%
Ballas et al. (2022) [24]	Selected 5 s heart-sound duration, data augmentation	-	1D CNN	73.7%
Our Method (PhysioNet Challenge 2016)	Selected 5 s heart-sound duration	Extracted MFCC features	K-NN, ANN, RF, and SVM	95.78%, 94.16%, 93.47%, and 86.19%
Our Method (PhysioNet Challenge 2022)	Selected 5 s heart-sound duration	Extracted MFCC features	K-NN, RF, ANN, and SVM	76.31%, 68.76% 65.18%, and 58.81%

## Data Availability

The dataset used in this study can be accessed on the PhysioNet website at https://physionet.org/challenge/2016/ (accessed on 5 May 2021) and https://physionet.org/content/circor-heart-sound/1.0.3/ (accessed on 9 September 2022).
